# Comment on ‘Oncological surgery follow-up and quality of life: meta-analysis’

**DOI:** 10.1093/bjs/znad367

**Published:** 2024-02-19

**Authors:** Yi Liu, Chenyu Chu, Qing Li, Yin Zhou

**Affiliations:** Medical Cosmetic Center, Chengdu Second People’s Hospital, Chengdu, Sichuan, China; Medical Cosmetic Center, Chengdu Second People’s Hospital, Chengdu, Sichuan, China; Medical Cosmetic Center, Chengdu Second People’s Hospital, Chengdu, Sichuan, China; Department of Orthopedics, Chengdu Second People’s Hospital, Chengdu, Sichuan, China; Medical Cosmetic Center, Chengdu Second People’s Hospital, Chengdu, Sichuan, China

The review valuably consolidates evidence suggesting less-intensive follow-up strategies after oncological surgery do not adversely impact health-related quality of life (HRQoL), emotional well-being or patient satisfaction^1^. However, some elements merit closer consideration. First, the heterogeneity in the types of cancers and treatments reported among the studies may lead to inconsistencies in how different patient populations respond to varied follow-up intensities. For example, breast cancer patients might have different concerns and psychological stresses compared to prostate cancer patients. Second, the nature of ‘less-intensive’ follow-up strategies is not clearly defined. These could range from less-frequent clinical visits to lesser degrees of psychological and emotional support. This broad definition may have potentially influenced the authors’ findings. Third, the metrics used for measuring HRQoL, emotional well-being and patient satisfaction can vary significantly. Standardizing these measurements might reveal more nuanced results. A solution to these concerns could be designing more specific studies for each cancer type, and investigating the role of individualized follow-up strategies. Digital solutions, like telehealth and patient-reported outcome measures (PROMs), might offer a means to provide continuous, individualized follow-up care. Furthermore, conducting more granular analyses of how patient satisfaction is influenced by different aspects of the follow-up approach (for example, frequency of clinical visits, emotional support provided) could be beneficial. While the paper’s findings offer a new perspective on oncological follow-ups, a closer, more patient-centred evaluation might further optimize postoperative surveillance to better cater to the unique needs of each cancer patient.
